# SARS-CoV-2 in Kidney Transplant Patients: A Real-Life Experience

**DOI:** 10.3389/fmed.2022.864865

**Published:** 2022-03-28

**Authors:** Biagio Pinchera, Lorenzo Spirito, Lucia Ferreri, Roberto La Rocca, Giuseppe Celentano, Antonio Riccardo Buonomo, Maria Foggia, Riccardo Scotto, Stefano Federico, Ivan Gentile, Rosa Carrano, “Federico II” COVID-19 Team, Amicone Maria

**Affiliations:** ^1^Department of Clinical Medicine and Surgery, University of Naples “Federico II”, Naples, Italy; ^2^Department of Neurosciences, Reproductive and Odontostomatological Sciences, Section of Urology, University of Naples “Federico II”, Naples, Italy; ^3^Department of Public Health, University of Naples “Federico II”, Naples, Italy

**Keywords:** kidney transplant, SARS-CoV-2, COVID-19, transplant, immunosuppression

## Abstract

**Background:**

The COVID-19 pandemic has significantly impacted the management of solid organ transplant recipients and on clinical evolution in post-transplantation. Little is known on the impact of SARS-CoV-2 infection in these patients. The severity and lethality of this disease in solid organ transplant patients are higher thanin the general population. This study aims to describe clinical characteristics of SARS-CoV-2 infection in solid organ transplant recipients followed in our center.

**Methods:**

In this observational study, we enrolled all kidney transplant recipientsattending the A.O.U. Federico II of Naples from March 2020 to January 2021. For each patient we evaluated the epidemiological and clinical characteristics as well as outcome.

**Results:**

We enrolled 369 kidney transplant patients (229, male, 62%). Of these, 51 (13.8%) acquired SARS-CoV-2 infection and 29 showed symptomatic disease. Of the 51 patients with the infection, 48 (94.11%) had at least one comorbidity and such comorbidities did not constitute a risk factor for a more severe disease. Hospitalization was necessary for 7 (13.7%) patients. Of these, 2 required low-flow oxygen supplementation, 3 non-invasive/high flow ventilation and 2 invasive ventilation. Finally, 2 patients died.

**Conclusions:**

Our study shows a lower mortality and hospitalization rate compared to figures available in the literature (4% vs. 13–30% and 14% vs. 32–100%, respectively). Furthermore, the comorbidities examined (hypertension, dyslipidemia, and diabetes) did not constitute a risk factor for a more severe disease condition in this patient category. Further studies with larger sample size are necessary to confirm these data.

## Introduction

The COVID-19 (CoronaVIrus Disease-19) pandemic has significantly impacted the management of solid organ transplant patients and the clinical evolution in post-transplantation, notably by reducing the activity of transplant centers. Currently, this category of patients is considered to be at greater risk for developing a severe course of COVID-19 disease (1). Most studies show a high risk of developing a severe form of the disease and a high lethality in solid organ transplant patients (2). In fact, in SOT (Solid Organ Transplant) recipients, the reported lethality for COVID-19 ranges from 13 to 30% (3) and the hospitalization rates range from 32 to 100% (4–6). Several studies showed a high hospitalization rate among kidney transplant patients, about 70% undergoing hospitalization and of hospitalized patients, about 25% requiring mechanical ventilation (7, 8). Therefore, also in Europe, data on the COVID-19 mortality rate among kidney transplant patients range between 19 and 50% (9, 10). Several studies evaluated the clinical characteristics of immunocompromised patients with SARS-CoV-2 (Severe Acute Respiratory Syndrome-CoronaVirus-2) infection, comparing them with the general population affected by this infection. From these studies it emerged that SOTs infected with SARS-CoV-2 had more frequently diabetes, cardiovascular disease, hypertension, respiratory disorders and more often needed hospitalization and intensive care, showing a higher lethality (11). Moreover, a systematic review and meta-analysis of SOT recipients with SARS-CoV-2 infection, enrolling 2,772 SOT recipients, showed that the majority (81%) needed hospitalization (12–14). However, the impact of SARS-CoV-2 infection in solid organ transplant patients is not fully understood and data on this topic are still scarce and scanty. This study aims to describe clinical characteristics of SARS-CoV-2 infection in solid organ transplant recipients followed in our center.

## Materials and Methods

We conducted an observational retrospective cohort study. We enrolled kidney transplant patients attending the A.O.U. Federico II of Naples and followed up from March 2020 to January 2021. Patients underwent regular rhino-oropharyngeal swabs for health surveillance or for suspected COVID-19 symptoms. In these patients, we evaluated rate of SARS-CoV-2 and of COVID-19 disease. Diagnosis of SARS-CoV-2 infection was defined as positivity to the rhino-oropharyngeal swab for SARS-CoV-2 RNA research by reverse transcription - polymerase chain reaction (RT-PCR). To describe the clinical status of SARS-CoV-2 infected patients we used the NIAID ACTT-1 (National Institute of Allergy and Infectious Diseases Adaptive COVID-19 Treatment Trial-1) Clinical Status Ordinal Scale (15). Based on this score, we classified each patient with the infection into one of eight categories: (1) Not hospitalized, no limitations on activities; (2) Not hospitalized, limitation on activities, and/or requiring home oxygen; (3) Hospitalized, not requiring supplemental oxygen and no longer requires ongoing medical care (if hospitalization extended for infection-control purposes); (4) Hospitalized, not requiring supplemental oxygen; requiring ongoing medical care (COVID-19 related or otherwise); (5) Hospitalized, requiring supplemental oxygen; (6) Hospitalized, on noninvasive ventilation or high-flow oxygen devices; (7) Hospitalized, on invasive mechanical ventilation or ECMO; (8) Death (15). In addition, for patients with COVID-19 disease, we also used the Henry Ford Hospital (HFH) COVID-19 severity scoring system to distinguish mild, moderate, and severe forms of the disease (16). Mild disease was defined as patients who had normal chest radiography and SpO2 of ≥94% without the need for supplemental oxygen. Moderate disease patients were those who had abnormal chest radiography, SpO2 of <94% and needing between 1 and 5 liters/min supplemental O2. Patients with severe disease were defined by abnormal chest radiography, SpO2 of <94% and requiring ≥6 liters/min of O2 (16). For each patient we evaluated epidemiological and clinical characteristics, laboratory and radiological data, the need for hospitalization and access to the ICU (Intensive Care Unit), the type of immunosuppressive treatment and the changes of immunosuppression during SARS-CoV-2 infection, the treatment for SARS infection-CoV-2 and the outcome. For each patient we evaluated SARS-CoV-2 IgG (Roche Diagnostics GmbH, Mannheim, positive threshold > 15 BAU/ml). Furthermore, we assessed the risk of co-infections. Data are presented as mean and SD or median and interquartile range (IQR), in case of Gaussian or non-Gaussian distribution, respectively. For correlation analysis, Pearson or Spearman tests were used for data distributed in Gaussian or non-Gaussian fashion, respectively. Continuous variables are compared by Student's *t*-test or Mann-Whitney *U*-Test, as parametric or non-parametric test, respectively. The p-value for statistical significance was set at <0.05 for all the tests. The odds ratio analysis was conducted to evaluate and measure possible risk factors for more severe disease evolution. In particular, age, sex, comorbidities and immunosuppressive therapy were assessed and compared, it did not adjust for confounders. The study was conducted in compliance with the Declaration of Helsinki and the principles of good clinical practice. The study was exempt from approval from an ethics' board.

## Results

We enrolled 369 kidney transplant patients (229, male, 62%) with a median age of 49 years (IQR, 18–86). Of these, 51 (13.8%) became infected with SARS-CoV-2 during the period of the study. Anagraphic and clinical features of these patients are reported in Tables 1, 2. Only 17/51 (33.3%) SARS-CoV-2 infected patients had positive anti-SARS-CoV-2 IgG antibodies performed 14–21 days after the onset of symptoms. Of the 51 SARS-CoV-2 infected patients, 29 (56.9%) showed COVID-19 (Tables 1, 2). The most frequent symptoms were fever and cough (Table 1). Seven of the 29 (13.7%) patients were admitted to hospital. Of these seven patients, two required low-flow oxygen supplementation, three non-invasive/high flow ventilation and two invasive ventilation. In relation to the Henry Ford Hospital (HFH) COVID-19 severity scoring system, we distinguished 22 mild (75%), two moderate (7%) and five severe (18%) forms in the 29 patients. Of the 51 patients with the infection, 48 (94.11%) had at least one comorbidity. However, comorbidities did not constitute a risk factor for a more severe disease condition [OR: 1.1, 95 CI (0.40–2.2); *p*: 0.480] (Tables 1, 2). We compared and evaluated SARS-CoV-2 infected patients with diabetes vs. non-diabetic patients, assessing the risk of evolving to a severe form of COVID-19 related disease [OR: 1.2, 95 CI (0.85–1.7); *p*: 0.240]. We also compared and evaluated patients with SARS-CoV-2 infection with cardiovascular disease vs. patients not affected by this condition, evaluating the risk of evolution toward a severe form of COVID-19 related disease [OR: 1.1, 95 CI (0.70–1.4); *p*: 0.290]. Twenty patients received therapy for COVID-19. In details, 19 received steroid therapy, 16 low molecular weight heparin, two Remdesivir. All patients with symptoms underwent modifications of immunosuppressive therapy (Table 1). In detail, at baseline, most patients were receiving calcineurin inhibitor (CNI) (92%) and corticosteroids (96%) at the time of the diagnosis of the infection. Antimetabolite (azathioprine and mycophenolate mofetil) were used in 49%, while mTOR (mammalian Target Of Rapamycin) inhibitors were in 18% of cases. With respect to patients with a moderate-severe form of the disease, calcineurin inhibitors (CNI), corticosteroids and antimetabolite were used in 100, 85, and 57%, respectively, while mTOR inhibitors were used by no patient with a moderate—severe form of the disease [OR: 1.27, 95 CI (0.60–1.8); *p*: 0.097]. Regarding the therapeutic management of the infection, the first step was the reduction of immunosuppressive therapy, which consisted in the reduction or suspension of antimetabolites in the case of moderate forms. In the case of severe forms of the disease, all immunosuppressive therapy was suspended, except for the steroid therapy. We observed 9/51 (17.6%) bacterial co-infections among patients with COVID-19: four urinary tract infections, three pneumonia and two sepsis. Only one patient experienced acute organ rejection. Finally, two patients died.

**Table 1:** Anagraphic and clinical features of enrolled kidney transplant patients with SARS-CoV-2 infection (*n* = 51).

**Age (median, IQR)**	**50 (18–71)**
**Gender:**	
M	41 (80.4%)
F	10 (19.6%)
**Patients with infection:**	
Asymptomatic with infection	22 (43.14%)
COVID-19	29 (56.86%)
Asymptomatic	22 (43.14%)
M	20 (90.9%)
F	2 (9.1%)
**Symptoms:**	
Fever	19 (65.5%)
Cough	12 (41.3%)
Asthenia	8 (27.6%)
Dyspnea	10 (34.5%)
Anti SARS-CoV-2 antibodies	17 (33.3%)
Comorbidities in patients with SARS-CoV-2 infection:	51
Hypertension	48 (94.1%)
Dyslipidemia	26 (50.9%)
Diabetes	7 (13.7%)
Anemia	13 (23.7%)
Ischemic heart disease	1 (1.96%)
Therapy for COVID-19:	29
Modifications of immunosuppressive therapy	20 (69%)
Steroid therapy	19 (65.5%)
Low molecular weight heparin	16 (55.1%)
Remdesivir	2 (6.8%)

**Table 2:** Anagraphic clinical features of patients with SARS-CoV-2 infection: asymptomatic vs. COVID-19.

	**Patients with SARS-CoV-2 infection**		***p*** **-value**
	**Asymptomatic** ***n*** **= 22 (43.14%)**	**COVID-19** ***n*** **= 29 (56.86%)**	
Age, years (median, IQR)	49 (26–70)	52 (18–71)	0.657
**Gender:**			
M	20 (90.9%)	21 (72.4%)	0.748
F	2 (9.1%)	8 (27.6%)	
**Comorbidities:**			
Hypertension	20 (90.9%)	28 (96.5%)	0.284
Dyslipidemia	10 (45.5%)	16 (55.2%)	0.310
Diabetes	3 (13.6%)	4 (13.7%)	0.540
Anemia	6 (27.3%)	7 (24.1%)	0.620
Ischemic heart disease	0	1 (3.4%)	0.218
**Immunosuppressive therapy:**			
Corticosteroids	21 (95%)	28 (96.5%)	0.620
Calcineurin inhibitor	20 (90.9%)	27 (93%)	0.244
Antimetabolite	9 (40.9%)	15 (51.7%)	0.186
mTOR inhibitors	8 (36.3%)	5 (17.2%)	0.112
Time from transplant to diagnosis of SARS-CoV-2 infection in months (median, IQR)	132 (7–420)	84 (7–264)	0.120

## Discussion

In our study we showed that the rate of SARS-CoV-2 infection was higher than that of the general population (13 vs. 2.6%) (1). In addition, we noted that the most majority of our patients were males while no risk factor for infection was identified.

Moreover, it was observed that only 33% of patients with infection had an anti-SARS-CoV-2 IgG serology. This finding is probably related to the characteristic immunosuppression of solid organ transplant patients which could suppress the production of an effective antibody response. However, no correlation was observed between the time from transplantation and the risk of infection [OR: 1.2, 95 CI (0.90–1.4); *p*: 0.190]. In this way, it could have been hypothesized that patients with a more recent transplant were more at risk of contracting the infection, given the more pronounced immunosuppression in the first months after transplantation. However, among infected patients, those ones with a symptomatic disease showed a trend toward a shorter time from transplantation to symptoms than those with an asymptomatic infection [OR: 1.1, 95 CI (0.50–1.7); *p*: 0.090].

Regarding the symptoms, in our cohort, kidney transplant patients with SARS-CoV-2 infection showed a high rate of symptomatic disease (56.9%). However, symptoms were mild in most cases (75%) and similar to those observed in non-transplant patients with SARS-CoV-2 infection. Moreover, our study shows a lower rate of admission to hospital compared to the data in the literature (14% vs. 32–100%). We also observed a lack of correlation between comorbidities and the risk of developing COVID-19 [OR: 1.1, 95 CI (0.40–2.2); *p*: 0.480] (17, 18). In particular, in our study having type 2 diabetes mellitus as a comorbidity did not constitute a risk factor for a more severe evolution of COVID-19 related disease [OR: 1.2, 95 CI (0.85–1.7); *p*: 0.240]. Furthermore, our study also highlighted that patients with cardiovascular pathologies did not present an increased risk of evolution toward a severe form of COVID-19 related disease [OR: 1.1, 95 CI (0.70–1.4); *p*: 0.290]. Our results are in contrast with those reported in the literature (19, 20). However, due to the relatively small sample size, our observation needs to be confirmed.

In relation to the immunosuppressive therapy, it was observed that no patient who presented a moderate-severe form of the disease, received immunosuppressive therapy which includes an mTOR inhibitor at the time of the diagnosis of infection. This result might be interpreted at the light of the potential antiviral effects of mTOR inhibitors (21), although an antiviral effect against SARS-CoV-2 has never been demonstrated. The small number of patients enrolled, and the design of our study prevent to draw a definitive conclusion but do generate a hypothesis that should be tested in an *ad hoc* study.

We underline that we observed only nine bacterial co-infections (17.6%). This confirms once again, even in a subset of immunocompromised patients, that there is an excessive use of antibiotic therapy during COVID-19 (22–24).

Finally, in our study, the rate of episodes of acute organ rejection during SARS-CoV-2 infection was similar to that found in the literature (1.9 vs. 1%) (25) while the mortality rate was lower than that reported in the literature (4% vs. 13–30%). Indeed, while in Jager's study et al. there was a morality rate in kidney transplant patients equal to 19%, in our case the mortality rate was much lower, in particular equal to 4% in our case. Probably this data is to be considered within the age of the population considered, in fact in our case the median age was much lower than that of the population considered by Jager (49 vs. 71.7) (26). Probably also the reduced hospitalization rate found in our experience is to be attributed to the younger age of the transplanted population considered at our Center (26). Furthermore, the reduced mortality and hospitalization rates found in our experience could also be partly attributable to the type of immunosuppressive therapy found in our case. In fact, in our experience, only 49% of SARS-CoV-2 infected patients practiced immunosuppressive therapy with antimetabolites. As evidenced by the study by Goffin et al., the intensity of immunosuppressive therapy, in particular triple therapy vs. dual immunosuppressive therapy, significantly impacted the severe evolution of the disease and the risk of mortality (27, 28).

We acknowledge that our study presents several limitations: the small sample size, the retrospective and monocentric design. Furthermore, we did not correct for multiple testing and we did not adjust for confounders. The strength of our study is the real-life setting and the availability of several weapons that were not available at the time of the previous reports on the topic, such as the use of corticosteroids, antivirals or anticoagulants.

## Conclusion

In our real-life study conducted in kidney transplant patients with SARS-CoV-2 infection, we showed a lower mortality and admission rate compared to those available in the literature (4% vs. 13–30% and 14% vs. 32–100%, respectively). The potential role of mTOR inhibitors in the management of SARS-CoV-2 infection needs to be further investigated in future studies.

## Data Availability Statement

The raw data supporting the conclusions of this article will be made available by the authors, without undue reservation.

## Ethics Statement

The studies involving human participants were reviewed and approved by Federico II University Ethics Committee. The patients/participants provided their written informed consent to participate in this study.

## Federico II COVID-19 Team

Amicone Maria, Borrelli Francesco, Buonomo Antonio Riccardo, Cattaneo Letizia, Conte Maria Carmela Domenica, Cotugno Mariarosaria, Di Filippo Giovanni, Foggia Maria, Gallicchio Antonella, Gentile Ivan, Giaccone Agnese, Lanzardo Amedeo, Mercinelli Simona, Minervini Fulvio, Piccione Amerigo, Pinchera Biagio, Reynaud Laura, Salemi Fabrizio, Sardanelli Alessia, Schiano Moriello Nicola, Scordino Fabrizio, Scotto Riccardo, Stagnaro Francesca, Tosone Grazia, Viceconte Giulio, Zappulo Emanuela, and Zotta Irene.

## Author Contributions

BP: conceptualization and writing—original draft preparation. LS and AB: writing—review and editing. LF and MF: investigation. RR: data curation. GC: software. RS: methodology and formal analysis. SF: validation. IG: writing—original draft preparation and supervision. RC: conceptualization and supervision. All authors contributed to the article and approved the submitted version.

## Conflict of Interest

The authors declare that the research was conducted in the absence of any commercial or financial relationships that could be construed as a potential conflict of interest.

## Publisher's Note

All claims expressed in this article are solely those of the authors and do not necessarily represent those of their affiliated organizations, or those of the publisher, the editors and the reviewers. Any product that may be evaluated in this article, or claim that may be made by its manufacturer, is not guaranteed or endorsed by the publisher.
